# Overexpression of Aromatase Alone is Sufficient for Ovarian Development in Genetically Male Chicken Embryos

**DOI:** 10.1371/journal.pone.0068362

**Published:** 2013-06-28

**Authors:** Luke S. Lambeth, David M. Cummins, Timothy J. Doran, Andrew H. Sinclair, Craig A. Smith

**Affiliations:** 1 Murdoch Childrens Research Institute, Royal Children’s Hospital, Melbourne, Australia; 2 Department of Paediatrics, The University of Melbourne, Melbourne, Australia; 3 Poultry Cooperative Research Centre, Armidale, NSW, Australia; 4 CSIRO Animal, Food and Health Sciences. Australian Animal Health Laboratory, Geelong, Australia; Laboratoire de Biologie du Développement de Villefranche-sur-Mer, France

## Abstract

Estrogens play a key role in sexual differentiation of both the gonads and external traits in birds. The production of estrogen occurs via a well-characterised steroidogenic pathway, which is a multi-step process involving several enzymes, including cytochrome P450 aromatase. In chicken embryos, the aromatase gene (*CYP19A1*) is expressed female-specifically from the time of gonadal sex differentiation. To further explore the role of aromatase in sex determination, we ectopically delivered this enzyme using the retroviral vector RCASBP *in ovo*. Aromatase overexpression in male chicken embryos induced gonadal sex-reversal characterised by an enlargement of the left gonad and development of ovarian structures such as a thickened outer cortex and medulla with lacunae. In addition, the expression of key male gonad developmental genes (DMRT1, SOX9 and Anti-Müllerian hormone (AMH)) was suppressed, and the distribution of germ cells in sex-reversed males followed the female pattern. The detection of SCP3 protein in late stage sex-reversed male embryonic gonads indicated that these genetically male germ cells had entered meiosis, a process that normally only occurs in female embryonic germ cells. This work shows for the first time that the addition of aromatase into a developing male embryo is sufficient to direct ovarian development, suggesting that male gonads have the complete capacity to develop as ovaries if provided with aromatase.

## Introduction

Sex determination in birds, as in mammals, occurs through the inheritance of specialised sex chromosomes at the time of fertilization [1]. Although the exact mechanism of avian sex determination is still unclear, genes on one or both of the sex chromosomes must initiate sexual differentiation of the embryonic gonads into testes or ovaries, and eventually all other sexually dimorphic features. In the chicken and in all other birds, a ZZ male: ZW female sex chromosome system exists. Two hypotheses have been proposed for the mechanism of avian sex determination. The W (female) chromosome may carry a dominant-acting ovary determinant, or alternatively the dosage of a Z-linked gene might be required for male development [2].

Male and female chicken embryonic gonads are morphologically indistinguishable up until 6 days post-fertilisation (Hamburger-Hamilton stage 29–30). In ZZ males, bilateral testes develop, characterised by seminiferous cords (Sertoli cells together with prospermatogonia) that develop in the inner part of the gonad (the medulla). In ZW females, the left gonad rapidly grows into an ovary with a thickened outer cortex that is the site of folliculogenesis, while the right progressively regresses into a vestigial organ. In both the left and right gonads, cells of the medulla express the cytochrome P450 enzyme, aromatase (*CYP19A1*). Aromatase shows female-specific expression in early gonads from the time of gonadal sex differentiation [3]–[6], and along with several other enzymes, catalyses estrogen synthesis. In genetically female chicken embryos, estrogen is critical for normal ovarian development [7], [8] and is also important for secondary sexual characteristics, such as the generally cryptic colouration of female birds. At the same developmental stage that aromatase becomes active in female embryonic gonads, the conserved testis gene *SOX9* becomes active in male gonads. SOX9 is expressed in the embryonic testis cords, where by analogy with other vertebrates, it is thought to trigger differentiation of the Sertoli cell lineage. These two factors, aromatase in female and SOX9 in male, are therefore considered to be of fundamental importance in establishing either an ovary or a testis in chicken embryos [5], [9], [10].

The manipulation of estrogen levels in the early chicken embryo induces sex reversal. Injection of a single dose of an aromatase enzyme inhibitor at day 3.5 of development is sufficient to produce ZW female-to-male sex reversal, characterised by the formation bilateral testis that are capable of spermatogenesis and an external male-type phenotype [7]. However, although females treated with the aromatase inhibitor fadrozole developed testis-like gonads and masculine-type male genitalia at hatching, these birds showed no difference in body weights compared to untreated females and an increasing portion had normal ovarian follicles as they aged [11]. It has also been found that exposure to exogenous estrogen can transiently feminise genetically male embryos. Both gonads appear ovarian, but this effect is not permanent, with individuals reverting to a male phenotype within one year of hatching [12], [13]. Estrogen levels can also vary naturally. The henny feathering trait, whereby roosters have identical feathering to the hens, was originally described in the Sebright Bantam and was attributed to an inherited condition that resulted in increased extragonadal activity of aromatase [14], [15]. It was found that the aberrant aromatase expression in these animals was due an allelic mutation in the sequence directly flanking aromatase that resulted in the insertion of a retroviral derived promoter sequence [16], [17]. Taken together, these experiments highlight the critical importance of aromatase and estrogen in chicken ovarian development.

Although aromatase expression in chickens is a hallmark of the female program of development, experimental overexpression of aromatase leading to a gonad phenotype has not been reported in this or any other vertebrate species. The current study aimed to test if overexpression of aromatase alone in male embryos could affect gonad development. We found that ectopic overexpression of this enzyme was sufficient to induce a male-to-female gonadal sex-reversal in genetic ZZ embryos, characterised by the development of the left gonad into ovarian tissue. This phenotype also included a disruption in the expression of key male genes including DMRT1 and SOX9, and upregulation of ovarian development genes FOXL2 and RSPO1. Furthermore, genetically male germ cells followed a female developmental pattern and were capable of undergoing meiosis. These data indicate that the overexpression of aromatase alone is sufficient for ovary development in genetically male chicken embryos.

## Materials and Methods

### Ethics Statement

All experiments were carried out with respect for the principles of laboratory animal care and were consistent with the *Australian Code of Practice for the Care and Use of Animals for Scientific Purposes, 7^TH^ Edition 2004* and the *Prevention of Cruelty to Animals Act, Victoria 1986*. This included official approval from the Murdoch Childrens Research Institute Animal Ethics Committee (AEC # A627).

### Aromatase Overexpression

To assess the effects aromatase mis-expression in chicken embryos we used the avian retroviral vector RCASBP [18], [19]. To clone aromatase, RNA was extracted from a Hamburger-Hamilton stage 36 (HH36) ZW female chicken embryo using TRIZOL Reagent (Invitrogen). RNA was then DNAse treated using the DNA-*free*™ kit (Ambion) and cDNA was synthesized using the First Strand cDNA Synthesis Kit (Roche). The same sequence of the aromatase open reading frame that was previously cloned and tested in vitro [20] (Accession J04047) was PCR amplified from cDNA using forward primer 5′-ATACCAGAAACTTTGAATCCACTGA-3′ and reverse primer 5′-ATAGGATCCCCCTGTAATGTTAATGCTGATCC-3′ with an included *BamH*I site. The resultant 1544bp fragment was subcloned into the *Nco*I and *BamH*I sites of pSLAX-13. The aromatase sequence was then cloned into RCASBP/A proviral DNA on *Cla*I and its sequence confirmed by DNA sequencing. The viral DNA was then transfected into chicken fibroblastic DF1 cells using Lipofectamine 2000 (Invitrogen) and propagated for approximately 2 weeks. Immunostaining of aromatase and the viral protein p27 at this stage confirmed infection and ectopic aromatase expression *in vitro*. Recombinant virus was harvested from culture medium, concentrated by ultracentrifugation and titered as previously described [21]. High titre virus (at least 10?8 Infectious Units/mL) was injected into day 0 blastoderms, and eggs were sealed with parafilm and incubated at 37.5°C for the indicated time points. Control embryos were either not injected, or injected with RCASBP/A virus carrying GFP (RCASBP-GFP). These experiments were repeated at least three times and involved at least 50 embryos for each experiment. Embryos were harvested at embryonic days 5.5 (HH28), 7.5 (HH32), 10.5 (HH36) or 18.5 (HH44). For the majority of experiments, E10.5 embryos were examined as at this time point male and female gonads are well differentiated, including a clear asymmetry and development of a thickened outer cortex in the left gonad in female, and the appearance of defined seminiferous cords in males. Immunostaining tissues with p27 antibody, which detects a viral epitope, confirmed RCASBP infection.

### PCR Sexing

Infected and control embryos were dissected at indicated time points. For genetic sexing of embryos, a small piece of limb tissue was digested in PCR compatible Proteinase K buffer and the genomic DNA was used for rapid PCR sexing [22]. By this method, only females show a W-linked (female-specific) *Xho*I band. Amplification of 18S rRNA in both sexes served as an internal control.

### Immunofluorescence

Control and aromatase mis-expressing tissues were fixed for 15 minutes in 4% PFA/PBS at room temperature, prior to processing for tissue section immunofluorescence, as described previously [23]. At least three embryos per time point and/or treatment were examined. Briefly, 10 µm sections were cut on a cryostat, permeablised in PBS 1% Triton X-100 and blocked in PBS 2% BSA for 1 hour. Primary antibodies were either raised in-house (rabbit anti-chicken aromatase (1∶5000), rabbit anti-chicken DMRT1 (1∶5000), rabbit anti-chicken vasa homologue (CVH) (1∶6000), rabbit anti-chicken FOXL2 (1∶6000), rabbit anti-chicken R-Spondin1 (RSPO1) (1∶2000)), or were obtained commercially (rabbit anti-p27 (1∶1000); Charles River Services, goat anti-AMH (1∶1000); Santa Cruz, mouse anti-fibronectin (1∶500); Serotec, goat anti-GFP (1∶500); Rockland, rabbit anti-mouse SCP3 (1∶700); Novus Biologicals). Alexa-fluor secondary antibodies were used (donkey or goat anti-rabbit, mouse or goat 488 or 594; Molecular Probes). Sections were counterstained with DAPI.

### Histology

Histology tissues were fixed overnight in Bouin’s fluid (Sigma), dehydrated in a tissue processor, embedded in paraffin and cut on a microtome at 4 µm. Sections were then stained in haematoxylin and eosin prior to mounting and imaging (Biomedical Sciences Histology Facility, University of Melbourne).

## Results

### Overexpression of Aromatase in Chicken Embryonic Gonads

**Figure 1:**
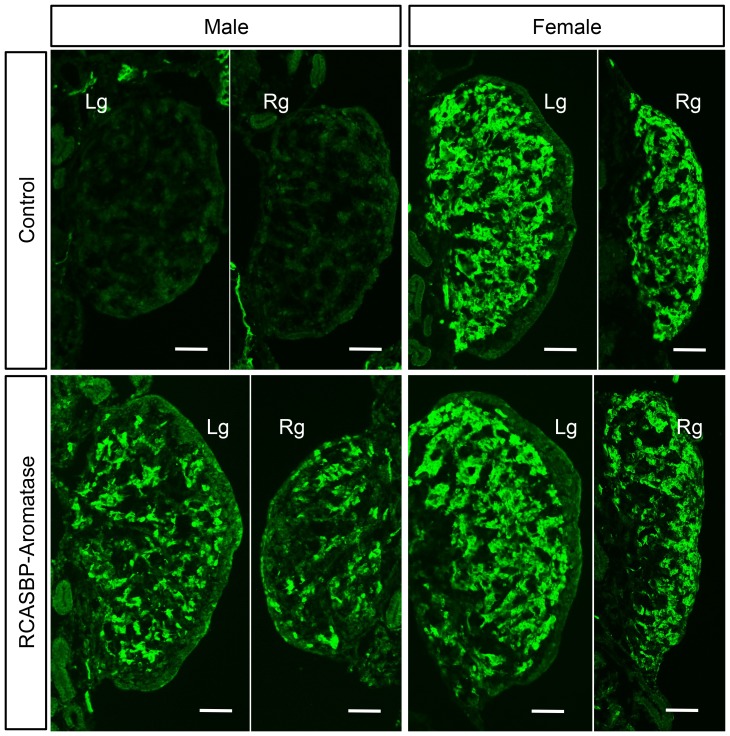
Ectopic expression of aromatase in embryonic gonads. RCASBP-Aromatase virus was injected into blastoderms and aromatase expression was assessed at embryonic day 10.5 by immunostaining (10×magnification). For each, the left (Lg) and right (Rg) gonads are shown with aromatase staining (green). No expression was detected in control males, while control females showed robust aromatase expression in the medulla. RCASBP-Aromatase infected embryos showed gonadal aromatase expression in both males and females.

For the *in ovo* overexpression of aromatase, we developed an RCASBP retroviral vector encoding the open reading frame sequence for this gene. DF1 cells were transfected with plasmid DNA of the recombinant RCASBP-Aromatase vector and overexpression of aromatase protein was confirmed by immunostaining with an anti-aromatase antibody (data not shown). To test the activity of this virus *in ovo*, chicken blastoderms were injected with concentrated recombinant virus and at embryonic day 10.5 (E10.5), embryos were dissected and gonads were analysed for aromatase expression by immunofluorescence (Figure 1). Staining of the left and right gonads of female controls indicated typical aromatase expression in the gonadal medulla, and as expected, no aromatase expression was detected in control male gonads. In male embryos infected with RCASBP-Aromatase, expression was evident in both the left and right gonads in an expression pattern that resembled that of a control female. Although this clearly demonstrated overexpression of aromatase in the male gonads, the level was somewhat lower than the control female. In female embryos infected with RCASBP-Aromatase, strong aromatase expression was observed at a similar level to the control female (Figure 1).

### The Effect of Aromatase Overexpression on Embryonic Gonad Development

**Figure 2:**
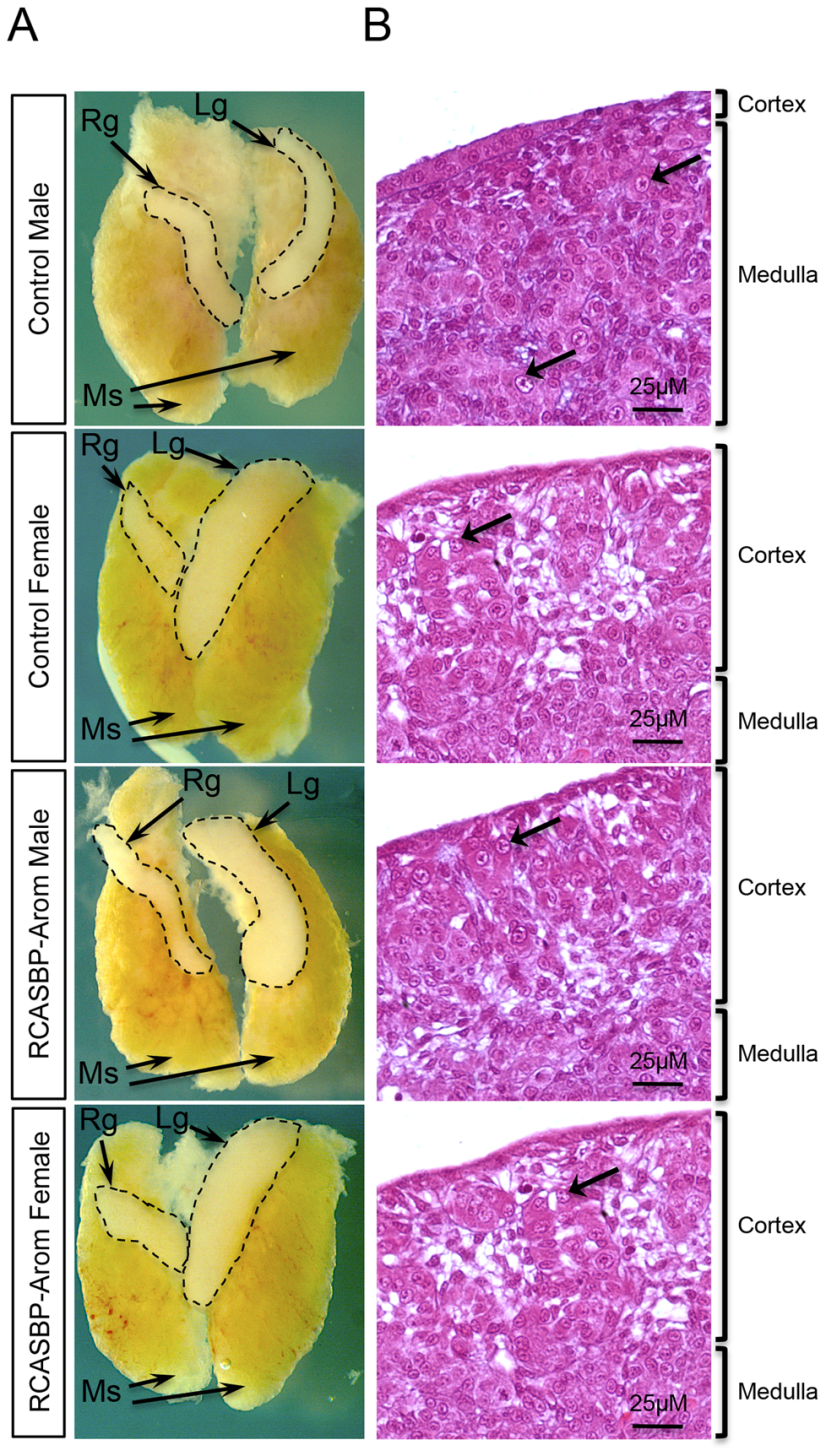
Phenotypic effects of aromatase overexpression in embryonic gonads. The urogenital systems of control and RCASBP-Aromatase virus infected E10.5 embryos. (A) Wholemount brightfield imaging (4×magnification) shows the mesonephric kidneys (Ms), and the right gonad (Rg) and left gonad (Lg), which is delineated by the black dashed lines. Gonadal asymmetry by enlargement of the left gonad of the RCASBP-Aromatase infected male is evident. (B) Haematoxylin and eosin staining of the left gonads of control and RCASBP-Aromatase injected embryos. The cortex and medulla regions for each are indicated and arrows highlight selected germ cells. Control males have a minimal cortex and large medulla, and very few germ cells, whereas the control female, and the RCASBP-Aromatase infected female and male have a thickened outer cortex and large numbers of germ cells.

To test the effect of aromatase overexpression on chicken gonad development, we analysed the gonads of E10.5 embryos for morphological changes. The urogenital systems of male and female controls showed normal gonad morphology for embryos at this stage of development, as shown by wholemount brightfield microscopy (Figure 2A). In particular, control males had cylindrical gonads with no obvious asymmetry, whereas control females had typical asymmetry characterised by a well developed and enlarged left ovary and a smaller regressing right gonad. RCASBP-Aromatase infected genetic ZW female embryos also exhibited a normal female gonad appearance. Genetic male embryos infected with RCASBP-Aromatase showed varying degrees of asymmetry, evidenced by the enlargement of the left gonad, which in some cases, was comparable to that of the female controls (Figure 2A). As an additional control, we also injected embryos with the EGFP expressing virus RCASBP-GFP which, as expected [21], showed high level GFP expression in both gonads of males and females with each showing normal development characterised by asymmetrical female gonads only (Figure S1).

The histology of E10.5 of aromatase overexpressing gonads was then analysed (Figure 2B). In control males, normal testicular morphology was evident, characterised by a thin outer cortex and well-developed seminiferous cords containing germ cells (arrows). In contrast, the control female left ovary had a thickened outer cortex with numerous germ cells. For the RCASBP-Aromatase infected embryos, the female showed normal gonad development whereas the left gonad of the male showed morphology that resembled a typical female structure. In particular, these males had a thickened outer cortex with a large number of germ cells (Figure 2B).

**Figure 3:**
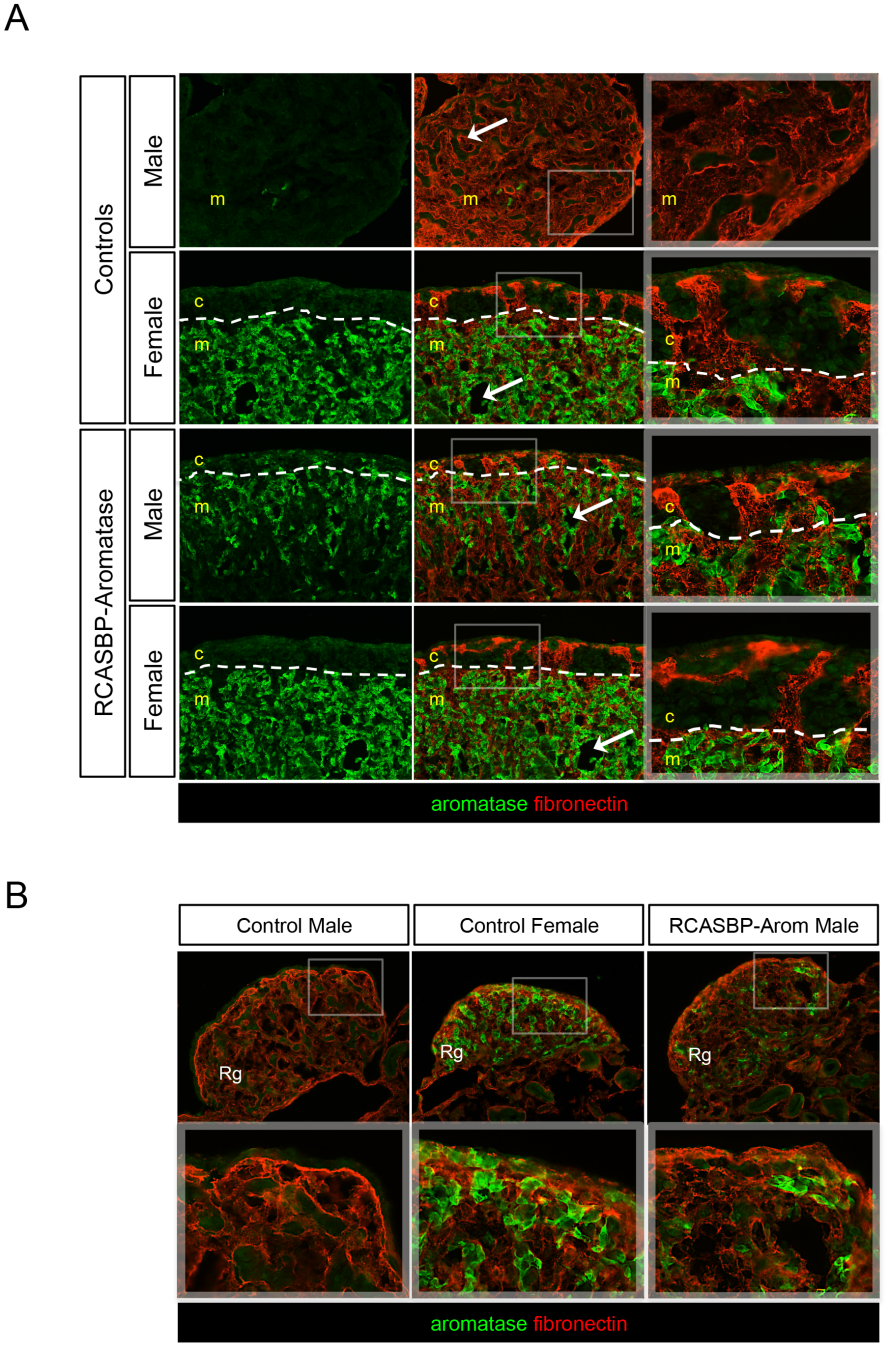
Immunostaining of feminised male embryonic gonads following aromatase overexpression. Gonads of control and RCASBP-Aromatase virus infected E10.5 embryos immunostained for aromatase (green) and fibronectin (red) (20×magnification). The medulla (m) and cortex (c) are indicated and boxed areas are shown adjacently as high power images. White dashed lines indicate medulla:cortex boundaries. (A) The left gonads of the control male and female show normal gonadal development. The male has a medulla with characteristic cord structures (arrows) and no aromatase expression. Females show strong aromatase expression and have a vacuolated medulla with lacunae (arrows) and a thickened outer cortex. The left gonad of an RCASBP-Aromatase infected male has strong aromatase expression and like the control female, features a thickened outer cortex, lacks cords in the medulla and contains lacunae (arrow). (B) The right gonads of a control males and females show normal gonad development. The right gonad of a male overexpressing aromatase shows lower aromatase expression compared to the control female, but appears to have disrupted formation of the testis cords.

To further analyse the effect of aromatase overexpression on male gonadal development, tissues were immunostained for both aromatase and the extracellular matrix glycoprotein fibronectin. In males, fibronectin delineates basement membrane and hence outlines testis cords, and in females it outlines medullary cords and delineates the fibronectin negative cords. No aromatase was present in left gonad of the control male, and fibronectin staining clearly outlined the structures of the highly organised seminiferous cords and cortex (Figure 3A). Strong aromatase expression was evident in the medulla of control female gonads with no expression in the cortex (Figure 3A). Fibronectin staining of the left gonads of RCASBP-Aromatase infected males revealed morphological features characteristic of female left gonads, such as a thickened outer cortex and disruption of the seminiferous cord structures of normal males. The overexpression of aromatase in these tissues also followed an expression pattern similar to that of the control female, i.e. localized to the medulla (Figure 3A). We also analysed the right gonads of both control and RCASBP-Aromatase infected males by immunostaining for aromatase and fibronectin. The control male right gonad had clearly defined seminiferous cords, whereas the female right gonad had a less organised medulla (Figure 3B). Despite lower levels of ectopic aromatase expression in the right gonad of an RCASBP-Aromatase infected male compared to the control female, the cord structures appeared to be somewhat disrupted and overall resembled the female structure.

### Time Course Analysis of Aromatase-mediated Sex Reversal

**Figure 4:**
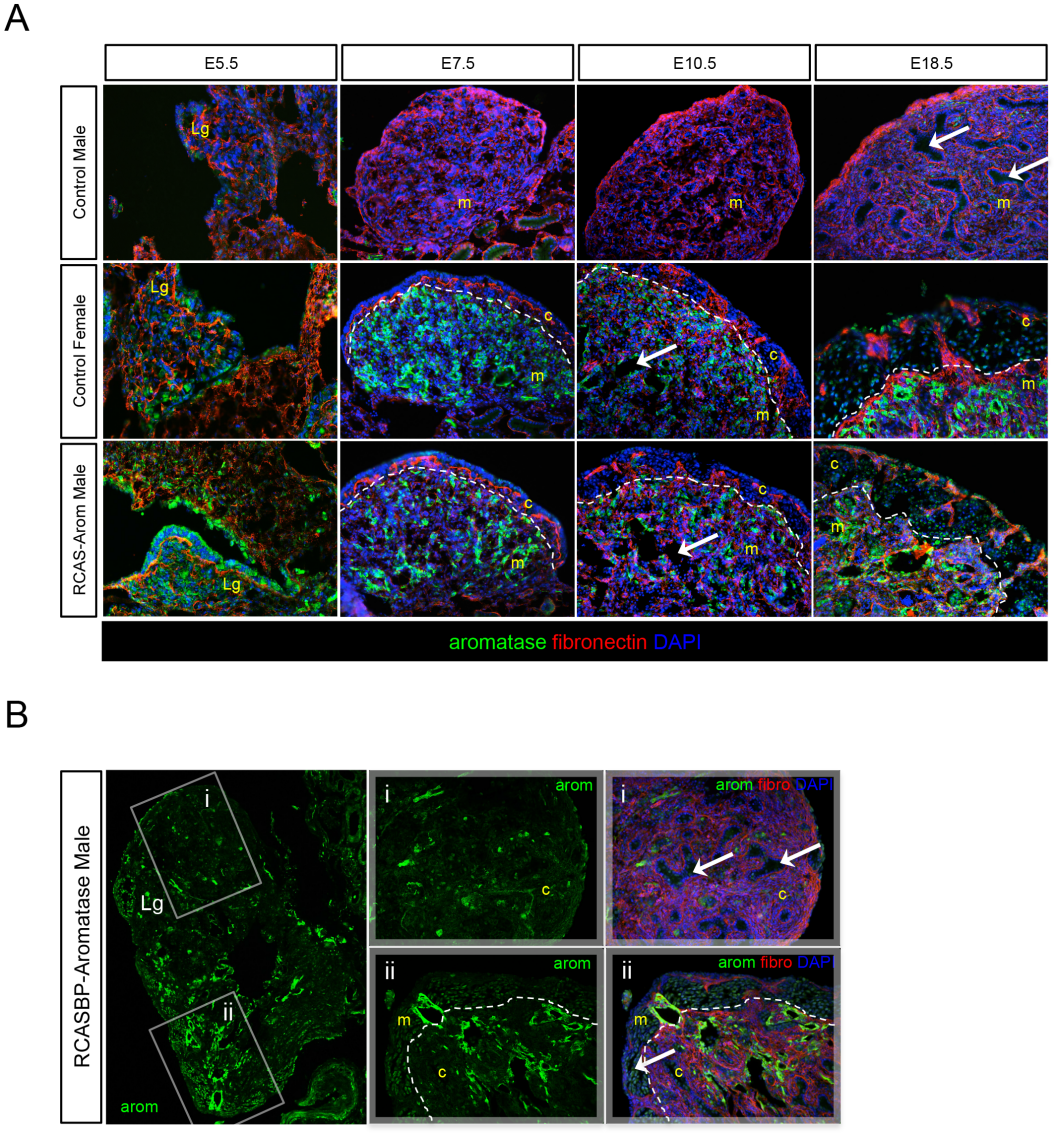
Progression of aromatase-mediated sex-reversal during gonad development. The gonads of control and RCASBP-Aromatase infected embryos immunostained for aromatase (green), fibronectin (red) and DAPI (blue) as indicated. The medulla (m) and cortex (c) are indicated and boxed areas are shown adjacently as high power images. White dashed lines indicate medulla/cortex boundaries. (A) Time course analysis of E5.5, E7.5, E10.5 and E18.5 left gonads (Lg) of control males and females, and RCASBP-Aromatase infected males (20×magnification). The control male features characteristic seminiferous cord structures within the medulla from E7.5 onwards. The control female and the RCASBP-Aromatase infected male have a thickened outer cortex, which increases in size progressively across all time points, and both have vacuolated medullary spaces (arrows). (B) RCASBP-Aromatase infected genetic male with an ovotestis. Left panel: a left gonad that contains areas of low (i) and high (ii) aromatase expression (10×magnification). (i) Normal male development indicated by the formation of cord structures (arrows), (ii) typical female morphology indicated by the formation of a thickened outer cortex (arrows) and disruption of cord structures (20×magnification).

The analysis of E10.5 RCASBP-Aromatase infected embryos showed a clear male to female sex-reversal. To further explore this phenotype throughout embryonic development, a time course analysis of aromatase overexpression was carried out. We examined gonads from E5.5 (just prior to sex differentiation), E7.5 (just after the onset of differentiation), E10.5 (mid-stage gonad development), and E18.5 (late stage embryos with well differentiated gonads). The expression of aromatase and fibronectin was monitored in the left gonads of control males and females, and RCASBP-Aromatase infected males by immunostaining. The control males showed an absence of aromatase expression across all time points, and from E7.5 onwards, fibronectin revealed well-defined cord structures (Figure 4A). The control female gonads showed aromatase expression at all time points tested, and the outer cortex increasingly thickened from E7.5 onwards. The RCASBP-Aromatase infected male gonads also showed aromatase expression at all time points tested and followed a pattern of localisation that closely resembled the control female. The development of a thickened outer cortex that increased in size across the time points was also very similar to the control female, as was the appearance of lacunae from E10.5 (Figure 4A).

Analysis of some immunostained RCASBP-Aromatase infected male gonads also showed that aromatase overexpression was in some cases variable throughout the gonad section. Inconsistent transgene expression from RCASBP is an observation that we have previously reported when using this vector for DMRT1 knockdown [24]. In the current study, areas of limited aromatase expression resulted in the formation of ovotesticular tissues (Figure 4B). In some individuals with partial feminization, the gonads tissues featured areas of normal male appearance with clearly defined cord structures, while others had localized thickening of the cortex, disruption of seminiferous cords, and the appearance of lacunae within the medulla. The appearance of these ovarian tissues correlated with the areas of higher aromatase expression.

### Overexpression of Aromatase Blocks the Testis-determining Pathway in Male Gonads

**Figure 5:**
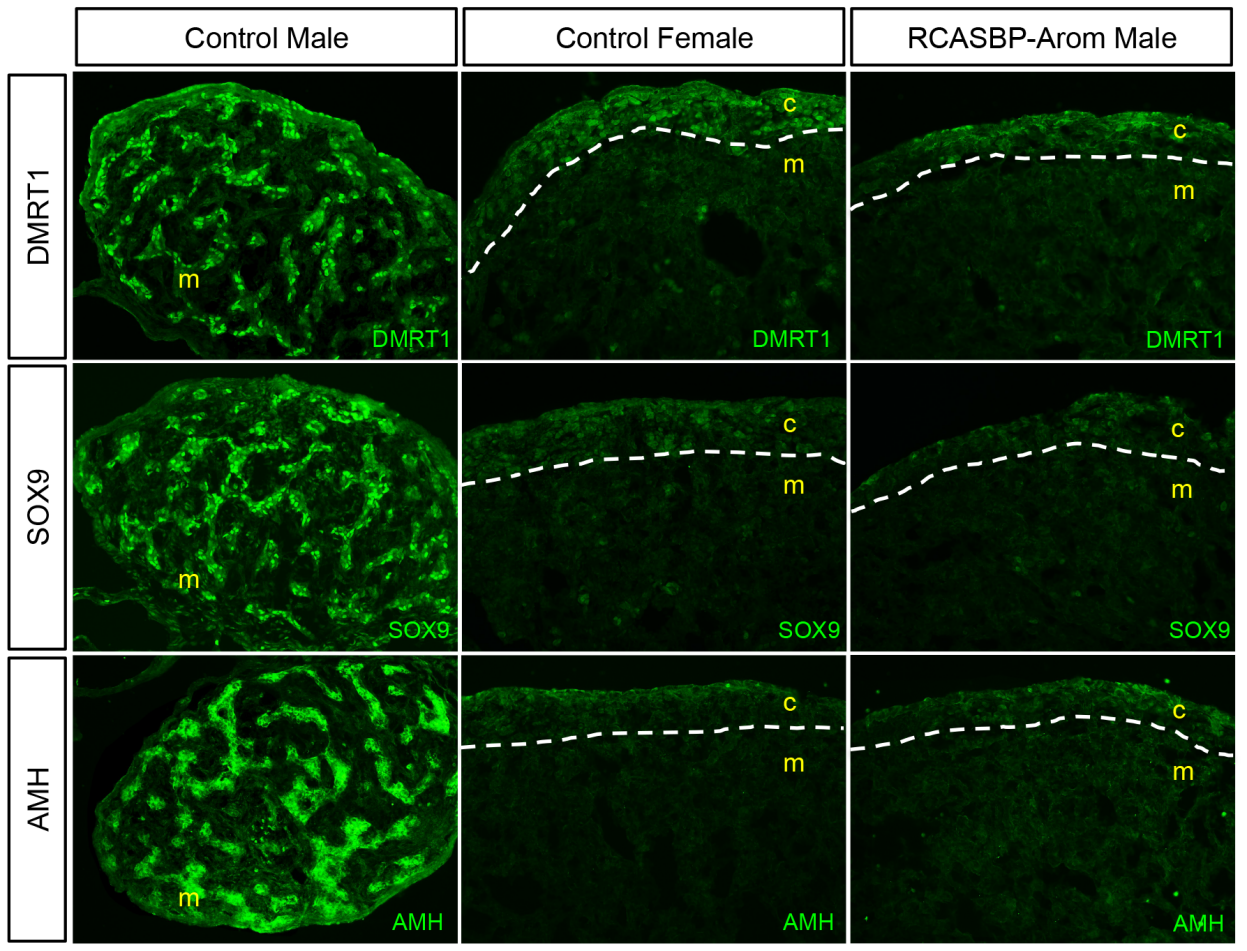
Expression of testis developmental genes in sex-reversed gonads. Left gonads of control and RCASBP-Aromatase infected embryos were immunostained for DMRT1, SOX9 and AMH (green) (20×magnification). The medulla (m) and cortex (c) are indicated and white dashed lines show the medulla/cortex boundaries. Control males show strong expression throughout testis cords for each, whereas both the control females and the sex reversed aromatase overexpressing males have little to no expression of these genes.

The expression of several key male-specific genes is required for testis development. To analyse the effect aromatase-induced sex reversal had on these genes, we monitored the expression of three key testis genes DMRT1, SOX9 and Anti-Müllerian hormone (AMH) by immunofluorescence on E10.5 gonads. Staining of the control males and females indicated normal expression levels and distribution of these proteins. For the males, strong expression was evident for DMRT1, SOX9 and AMH, and in each case this expression was confined to seminiferous cords (Figure 5). In contrast, very low level or no expression was evident in the females for each of these genes. In the RCASBP-Aromatase infected male gonads, the expression of all three of these genes was suppressed and showed expression patterns that were very similar to the female controls (Figure 5). In addition to the modulation of these key male genes in the left gonads, similar suppressive effects were also seen in the right gonads of sex reversed males (Figure S2). Taken together, these results showed that these three key testis development genes were not expressed in male gonads as a result of aromatase mis-expression, and their expression was indistinguishable from normal female gonads at this stage of development.

### Effect of Aromatase Overexpression on Female Pathway Genes

**Figure 6:**
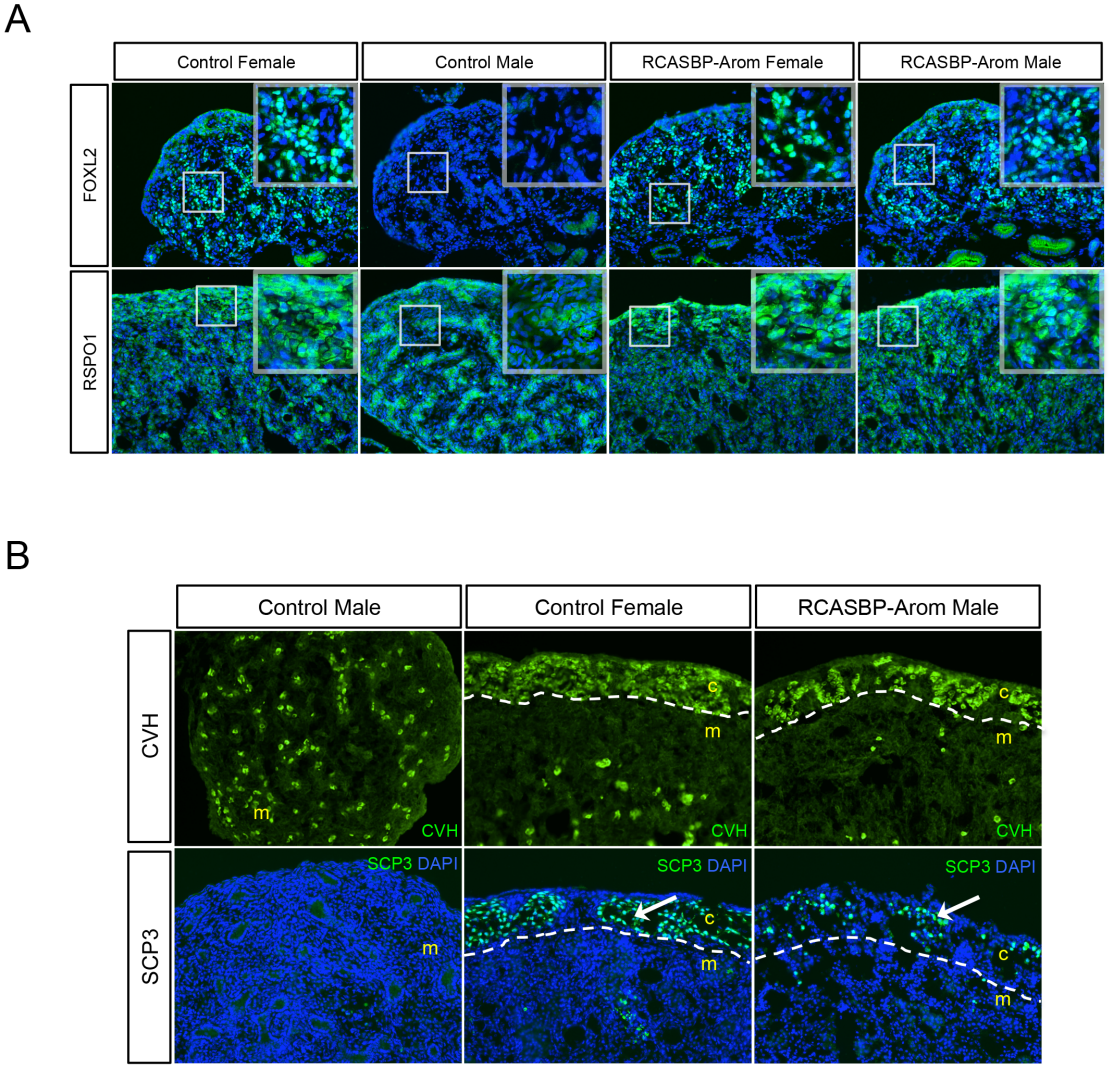
Expression of ovarian developmental genes and germ cell markers in sex-reversed gonads. Left gonads of control and RCASBP-Aromatase infected embryos were immunostained for FOXL2, RSPO1, CVH or SCP3 (green), and DAPI (blue) (20×magnification). The medulla (m) and cortex (c) are indicated and boxed areas are shown as high power images. White dashed lines indicate medulla/cortex boundaries. (A) E7.5 left gonads: FOXL2 expression in control and RCASBP-Aromatase infected females occurs throughout the gonad and is confined to the nucleus of positive cells. Background staining can be seen in the control male, whereas high levels of nuclear FOXL2 can been seen in the RCASBP-Aromatase infected male. E7.5 left gonads: RSPO1 expression in control and RCASBP-Aromatase infected females occurs throughout the gonad and is more intense in the cortex. Staining in the cords is evident in the control male, whereas the RCASBP-Aromatase infected male shows an expression pattern that is similar to the females. (B) E18.5 left gonads: The control male has CVH positive germ cells scattered throughout the medulla, whereas control female and the RCASBP-Aromatase male have only a few CVH positive germ cells in the medulla and large numbers localised within the outer cortex. No SCP3 positive cells are present in the control male. The female control and the RCASBP-Aromatase infected male have SCP3 positive germ cells in the outer cortex.

The expression patterns of ovarian development pathway genes FOXL2 and RSPO1 were then analysed by immunostaining left gonads. FOXL2 expression was monitored in E7.5 embryos as chicken gonads at this stage of development show robust female-specific expression [3], [25]. For this experiment, normal FOXL2 expression patterns were indicated by the male and female controls (Figure 6A). The female showed widespread nuclear expression throughout the cortex and medulla, whereas in the male no FOXL2 was detected. In RCASBP-Aromatase infected male and female E7.5 gonads, FOXL2 expression was similar to the control female. This included strong nuclear staining in both the cortex and medulla.

The expression of RSPO1 in aromatase overexpressing gonads was then analysed in E10.5 embryos, as female gonads from E10.5–E12.5 show significantly higher levels of expression compared to males [26]. Immunostaining for RSPO1 showed that a high level of expression was seen in the control female, particularly in the cortex. The control male showed a very different expression pattern with staining in the cords. In the RCASBP-Aromatase infected female and male gonads, RSPO1 expression followed a similar level and pattern as the control female, with higher expression in the cortex (Figure 6A). Taken together, these data showed that both ovarian developmental genes FOXL2 and RSPO1 were upregulated in response to aromatase overexpression in male gonads.

### Aromatase Overexpression Diverts Male Germ Cells to a Female Fate

To analyse the localization and replicative states of the germ cells in aromatase-induced sex-reversed male gonads, the expression of the germ cell marker chicken vasa homologue (CVH) and the meiotic germ cell marker SCP3 were monitored. For CVH, the control male gonad showed a scattering of positive cells throughout the medulla, whereas the control female had fewer cells in the medulla and large numbers throughout the cortex (Figure 6). As in control females, the germ cells of the RCASBP-Aromatase infected male gonads were mostly found in the cortex. Next, we determined if these mis-localized male germ cells were capable of entering meiosis, a process that normally only occurs late-stage females germ cells during embryonic development [27]. Immunostaining for the meiosis marker SCP3 in control males and females showed female-specific expression in E18.5 gonads (Figure 6). Although fewer in number compared to the control female, SCP3 positive germ cells were present in the cortex of the aromatase over-expressing sex-reversed male gonads, indicating that these germ cells not only showed a female-like distribution, but were also capable of undergoing meiosis.

## Discussion

This study shows that the over expression of a single steroidogenic enzyme, aromatase, is sufficient for female gonad development in genetically male embryos. The temporal and spatial expression patterns of the enzymes involved in steroidogenesis determine the ratio of androgens to estrogens in developing gonads [3]. Studies into the exact roles of these enzymes suggest that the lack of estrogen synthesis in males occurs due to the absence of both aromatase and 17β-Hydroxysteroid dehydrogenase (17β-HSD) [3]. These two enzymes are indispensable for the final steps of the steroidogenic pathway, responsible for the conversion of androgen substrates into active estrogens (estrone and 17β-estradiol) [28]. Although it has been shown experimentally that the direct manipulation of steroid hormone levels in chickens can cause sex-reversal [11]–[13], these effects are generally only transient. Nevertheless, these experiments highlight the fundamental role that estrogen and its production by aromatase play in females, however the effect of aromatase misexpression at the time of sexual differentiation was unknown.

We developed viral vectors for aromatase overexpression *in ovo* using RCASBP, which upon injection into blastoderm stage embryos allow for embryo-wide infection and transgene expression from the viral LTR promoter [21]. Initial analysis of E10.5 gonads by immunofluorescence showed that male embryos, usually negative for aromatase, showed high levels of overexpression of this protein, albeit at slightly lower levels compared to control females. Although this overexpression in infected males did not match the levels in normal females, analysis of the phenotype of E10.5 gonads revealed ovarian development in these genetic males. This macroscopic change in gonad morphology was reflected by histological analysis of the aromatase-overexpressing males, which showed that the gonad had developed a thickened outer cortex, a phenotype that closely matched the structure of the female left gonad at the same age. We have observed previously that embryos infected with RCASBP show varying degrees of transgene expression [24], so it was not surprising that the RCASBP-Aromatase infected individuals showed a variety of levels of feminisation, indicated by varying degrees of left gonad asymmetry (Figure 2A).

A deeper analysis of this sex reversal phenotype of aromatase overexpressing male gonads was provided by co-staining for fibronectin and aromatase, and by examining the gonads of embryos sampled at early, middle and late stages of development. These data showed that the extent of this sex reversal was sufficient to produce a morphology that was essentially identical to the normal female phenotype, and that this change occurred at the time of sex differentiation (E7.5) and was maintained in late-stage embryos just prior to hatching (E18.5). The retroviral method used here should deliver gene expression ubiquitously throughout the embryo, as we have seen for EGFP expression [21]. In the current study, we found that embryos infected with RCASBP-GFP virus showed strong EGFP expression throughout both male and female gonads, which in females included the cortex as well as the medulla (Figure S1). It was therefore interesting to find that overexpressed aromatase in males was limited to the parts of the gonad where it is normally expressed in females, and in females the levels of aromatase did not appear to be any higher than the female control. These findings suggest that a separate layer of control over both the location and level of aromatase expression may have been enforced. It was also found that the site of aromatase overexpression in gonad tissues was an important aspect of sex phenotype. In particular, variable or patchy localised aromatase expression produced an ovotestis phenotype (Figure 4B). In these gonads, the level of local estrogen production was sufficient to create regions with a thickened cortex and vacuolation of cords. These results highlighted the requirement for aromatase to be expressed throughout the gonad at sufficient or similar levels to the native expression seen in female gonads, and suggest that estrogen was acting locally.

Our data also indicated that aromatase expression (and hence presumably estrogen synthesis) was sufficient to suppress the testis pathway (DMRT1, SOX9 and AMH). However, it is unclear whether one or all of these genes were affected directly. The effects are most likely mediated via antagonism with DMRT1, which is the first of these genes to be expressed in males and is required for testis determination. In addition to the testis pathway genes, analysis of ovarian implicated genes FOXL2 and RSPO1 showed that the female molecular pathways were also affected by aromatase overexpression. Since FOXL2 is predicted to be an activator of aromatase [29] and hence upstream in the pathway, the observation that its expression was elevated in sex reversed male gonads suggests that either this assumption is incorrect, or that a feedback mechanism exists that allows FOXL2 expression to be induced. RSPO1, which acts through a separate pathway involving WNT4 and β-catenin, is important in vertebrate ovarian development and is active in the developing chickens [26]. The effect of estrogen on RSPO1 expression has already been demonstrated by drug-mediated inhibition of aromatase, which caused its expression in female gonads to be suppressed [26]. The current study therefore supports these observations, as we saw RSPO1 expression increase in response to aromatase-mediated sex reversal. Overall, the modulation of these key male and female gonad development genes indicated that aromatase overexpression in genetic males caused gonad feminisation at the both structural and molecular levels.

Aromatase mediated sex reversal also extended its effect to germ cells. The appearance of CVH positive cells in the cortex was in stark contrast to the expression pattern of germ cells in normal males, where they are characteristically far fewer in number and are scattered throughout the medulla. Furthermore, the finding that these mis-localised germ cells were positive for the meiotic marker SCP3 indicated that these sex-reversed genetic male gonads could potentially be reproductively viable as female gametes later in development. Indeed, it would be interesting to examine the effect of aromatase overexpression on hatched genetic male chickens to determine if the gonads are completely sex-reversed and able to develop into mature ovarian follicles capable of oocyte production, and if secondary sexual characteristics such as colouration would also be affected.

The data presented here clearly showed that the addition of aromatase alone into genetic male embryos was sufficient for female gonad development. However, in addition to aromatase, the final conversion of steroid intermediates into active estrogens also involves the catalytic activity of 17β-HSD. The expression of chicken 17β-HSD has been detected in the developing left and right gonads of females, but not in males [6]. A chicken cDNA copy of a *17β-HSD* sequence was cloned from female ovarian tissues, while its expression was not detected in equivalent male testis samples [30]. Although these and other studies show that males do not express 17β-HSD, these reports were focused on subtype 1 of this enzyme [6], [30], [31]. In mammals however, there have been up to fourteen 17β-HSD subtypes described so far [32]. Interestingly, 17β-HSD type-3 has been found to show testis-specific expression in mice [33] and abnormalities in its expression can lead to XY disorders of sexual development [34]. Although the chicken genome does not appear to encode any sequences that share any significant identity to human *17β-HSD type-3* (Human genome; Feb. 2009 GRCh37/hg19 assembly), recent data from our lab generated using RNA Sequencing on E4.5 gonads showed that chicken 17β-HSD type-4 was highly expressed males [35].

These findings suggest that despite an absence of 17β-HSD type-1 in male gonads, other subtypes may be present that are able to convert steroid hormone intermediates into active estrogens. Alternatively, it is possible that the role of 17β-HSD in estrogen synthesis is dispensable and aromatase can alone act to convert sufficient estrogens for female development. This may involve estrogens other that 17β-estradiol. For example, estrone is produced through the action of aromatase alone, acting on androgenic substrates without involvement of 17β-HSD [36]. Although estrone is considered a weak estrogen, it could be produced at sufficient levels in males over-expressing aromatase to induce the ovarian development reported here. In any case, the current study suggests that in chicken embryos, it is aromatase and not 17β-HSD that is the limiting factor in the production of sufficient estrogens needed to direct female gonad development. It is however, entirely possible that the effect of aromatase overexpression is transient and that in adult birds 17β-HSD (and/or other factors) might be required for complete gonadal sex reversal and to reach levels of fertility that match those of a normal female.

The basic mechanism of sex determination in chickens is likely to occur either through the action of dominant-acting female determinant carried on the W chromosome or by a Z-dosage mechanism (i.e. two copies for male development and one for female). The data presented here could support either modes of sex determination. Evidently, aromatase is an absolute requirement for female development and its addition alone in a male gonad is sufficient to override testis gene expression and drive ovarian development. Therefore, the factor that is leading to the eventual activation of aromatase could be a W-linked gene that is not expressed in male gonads. This gene could act directly or perhaps by inducing FOXL2, which is thought to activate aromatase [25], [37]. Alternatively, the dosage of a Z gene acting upstream of aromatase, such as DMRT1, has simply been overridden experimentally by the addition by this enzyme described here. It should be noted however, that a gonadal sex determination mechanism that relies upon an initial two-fold dosage system might be considered susceptible to perturbation, especially given the potency of aromatase.

Although the importance of aromatase in maintaining female gonadal development has been well documented, this study shows that its overexpression in a genetically male chicken embryo can also affect gonad phenotype. The data presented here indicates that regardless of the status of 17β-HSD in male gonads, aromatase alone is sufficient to induce ovarian development and male-to-female sex-reversal in chicken embryos. This work therefore builds upon previous studies that have identified the fundamental role that aromatase plays in the female program of development and implies that all other factors required to produce a female gonadal phenotype are already present in genetic male embryos.

## Supporting Information

Figure S1 **RCASBP-EGFP expression in E10.5 gonads.** RCASBP-GFP virus was injected into blastoderms and EGFP expression was monitored in dissected gonads. (i) wholemount fluorescence microscopy of male and female urogenital systems (4× magnification). The right gonad (Rg), left gonad (Lg) and mesonephric kidneys (Ms) are shown for each. Uninjected controls shown only background fluorescence, whereas injected embryos show strong EGFP expression. (ii) Immunofluorescence of EGFP expression in RCASBP-GFP injected male and female gonads. The medulla (m) and cortex (c) are indicated for each (20× magnification)
(TIF)

Figure S2 **Low magnification imaging of male gene expression in the left and right gonads of sex reversed embryos.** Control and RCASBP-Aromatase injected embryos were immunostained for DMRT1, SOX9, AMH and CVH expression (green). (i); Control males (ii); control females (iii); RCASBP-Aromatase injected males. The right gonad (Rg), left gonad (Lg) and mesonephric kidneys (Ms) are shown for each. 10× magnification.
(TIF)
