# Supplementary figures and images for: Overexpression of Aromatase Alone is Sufficient for Ovarian Development in Genetically Male Chicken Embryos
Source: PLoS One. 2013 Jun 28;8(6):e68362. doi: 10.1371/journal.pone.0068362 (PMC3695963; doi:10.1371/journal.pone.0068362)

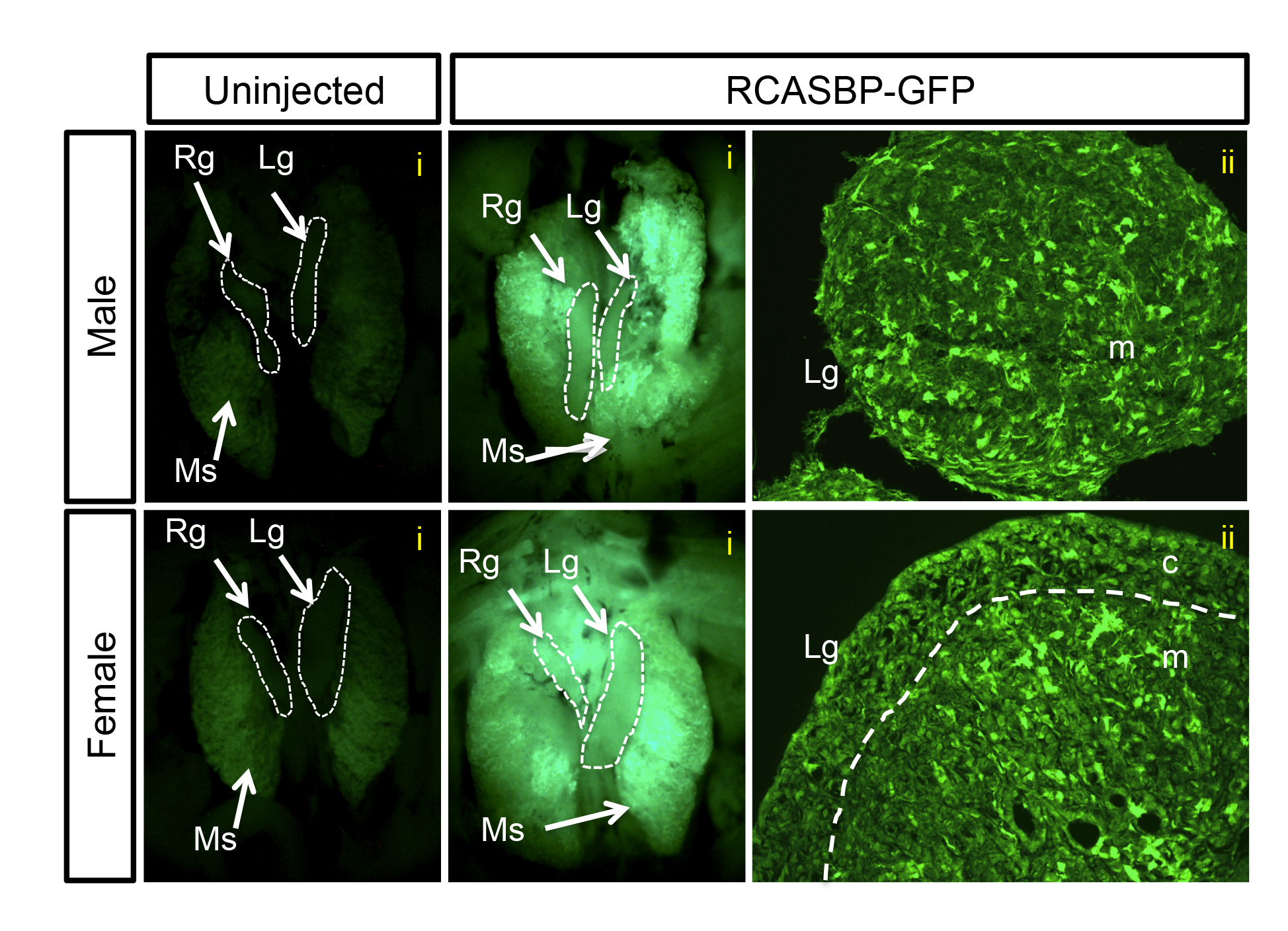

Supplement: Figure S1 — RCASBP-EGFP expression in E10.5 gonads. RCASBP-GFP virus was injected into blastoderms and EGFP expression was monitored in dissected gonads. (i) wholemount fluorescence microscopy of male and female urogenital systems (4× magnification). The right gonad (Rg), left gonad (Lg) and mesonephric kidneys (Ms) are shown for each. Uninjected controls shown only background fluorescence, whereas injected embryos show strong EGFP expression. (ii) Immunofluorescence of EGFP expression in RCASBP-GFP injected male and female gonads. The medulla (m) and cortex (c) are indicated for each (20× magnification) (TIF) [file pone.0068362.s001.tif]

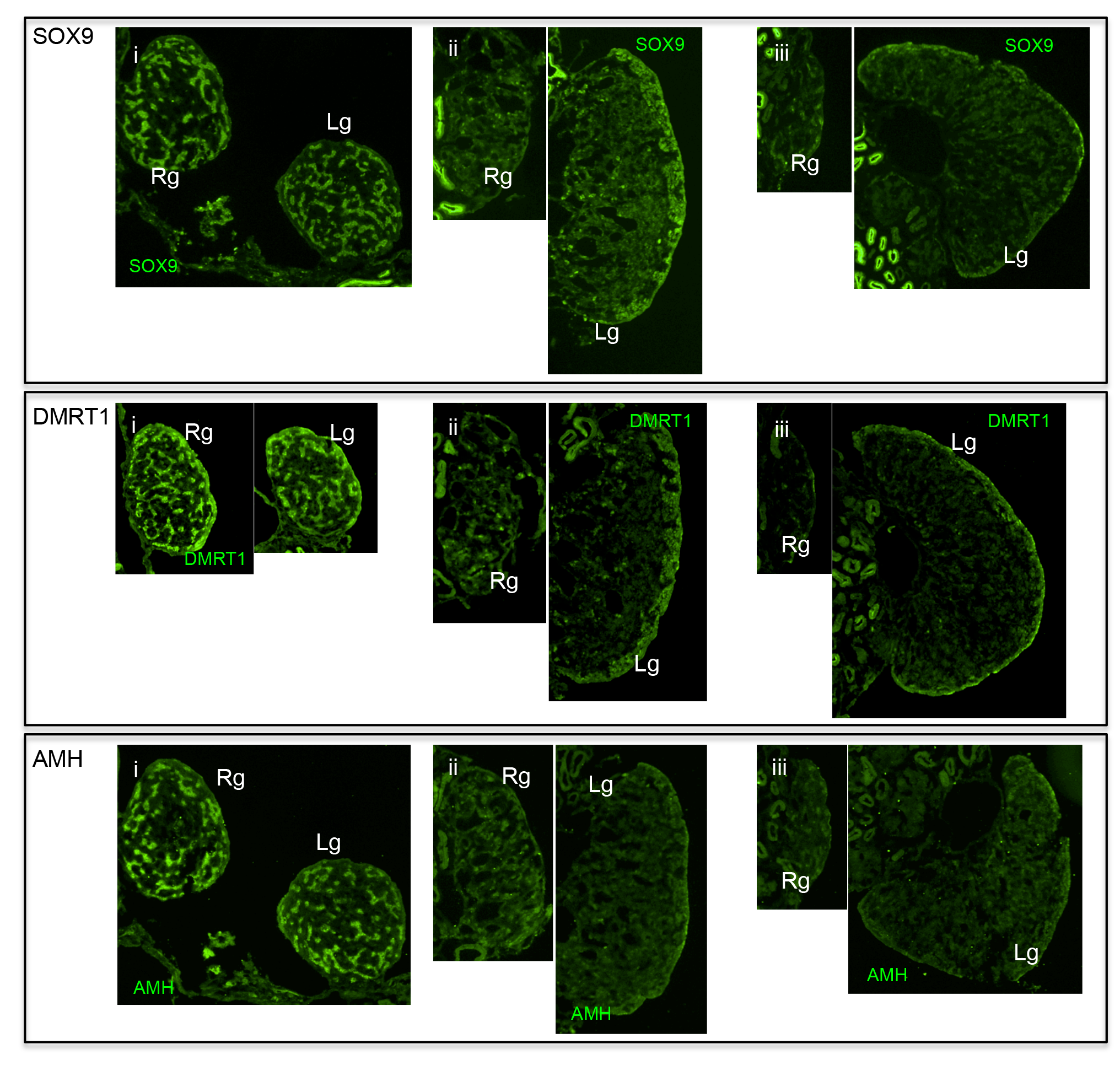

Supplement: Figure S2 — Low magnification imaging of male gene expression in the left and right gonads of sex reversed embryos. Control and RCASBP-Aromatase injected embryos were immunostained for DMRT1, SOX9, AMH and CVH expression (green). (i); Control males (ii); control females (iii); RCASBP-Aromatase injected males. The right gonad (Rg), left gonad (Lg) and mesonephric kidneys (Ms) are shown for each. 10× magnification. (TIF) [file pone.0068362.s002.tif]
